# Technical advances contribute to the study of genomic imprinting

**DOI:** 10.1371/journal.pgen.1008151

**Published:** 2019-06-20

**Authors:** Yuanyuan Li, Jinsong Li

**Affiliations:** State Key Laboratory of Cell Biology, Shanghai Key Laboratory of Molecular Andrology, CAS Center for Excellence in Molecular Cell Science, Shanghai Institute of Biochemistry and Cell Biology, Chinese Academy of Sciences, University of Chinese Academy of Sciences, Shanghai, China; University of Pennsylvania, UNITED STATES

## Abstract

Genomic imprinting in mammals was discovered over 30 years ago through elegant embryological and genetic experiments in mice. Imprinted genes show a monoallelic and parent of origin–specific expression pattern; the development of techniques that can distinguish between expression from maternal and paternal chromosomes in mice, combined with high-throughput strategies, has allowed for identification of many more imprinted genes, most of which are conserved in humans. Undoubtedly, technical progress has greatly promoted progress in the field of genomic imprinting. Here, we summarize the techniques used to discover imprinted genes, identify new imprinted genes, define imprinting regulation mechanisms, and study imprinting functions.

Mammals have two sets of chromosomes, one inherited from each parent. With the exception of postmeiotic germ cells, mammalian cells maintain a diploid genome during embryonic and postnatal development, and normally both alleles are equally expressed in cells. Intriguing observations from mouse genetic studies in the 1970s showed functional differences between paternal and maternal chromosomes in specific regions [1–3] and raised the possibility that the two sets of chromosomes are not functionally equivalent during embryonic development. One line of evidence supporting parent-of-origin effects on embryonic development came from Johnson’s observations of the mutant “hairpin-tail” mouse line, which carried a large, proximally located deletion in chromosome 17 [4]. He showed in utero the lethality of offspring receiving this deletion from a maternal parent, whereas paternal inheritance led to viable and fertile animals [4]. Similarly, mutant mice carrying chromosomal translocations (Robertsonian or reciprocal translocations) were used to generate mice with uniparental disomies (maternal or paternal duplication with corresponding deficiency) and determine the parent of origin for distinct chromosomal regions [5, 6]. Some of these disomic embryos displayed parental-specific lethality, suggesting the possibility “that haploid expression of particular maternal or paternal genes is important for normal mouse development” [6].

Several years later, in 1984, the most direct evidence of functional nonequivalence of mammalian parental genomes was provided by the Solter and Surani laboratories: reconstructed diploid embryos containing two maternal or two paternal genomes were generated through improved nuclear transfer technology [7–10]. Specifically, they placed a male or female pronucleus (isolated from a newly fertilized egg) in a host-fertilized egg from which the pronucleus had been removed to generate bipaternal (also known as androgenetic [AG]) or bimaternal (gynogenetic or parthenogenetic [PG]) embryos. However, the reconstructed bipaternal or bimaternal mouse embryos failed to survive; only the embryos with one maternal and one paternal genome produced viable and fertile offspring. The nuclear transfer experiments strongly suggested that genomic imprinting of parental genomes may be essential for complete embryogenesis. Meanwhile, a whole-genome screen of mouse with Robertsonian translocations led to identification of 13 subchromosomal regions in which both a maternal and paternal chromosome are required for normal development [3, 11].

Subsequent efforts were made to identify specific imprinted genes based on previous observations. Emergent techniques in mouse genetics (positional cloning, gene knockout, and allele-specific activity in hybrids) enabled researchers to distinguish between expression from maternal and paternal chromosomes, and the first three imprinted genes—insulin-like growth factor 2 receptor (*Igf2r*) [12], insulin-like growth factor 2 (*Igf2*) [13, 14], and *H19* (a noncoding RNA) [15]—were identified in 1991. To date, approximately 150 imprinted genes have been identified in mice, most of which map to the 13 subchromosomal regions identified in the 1980s. The majority of imprinted genes are located in clusters that contain several protein-coding genes and at least one noncoding RNA [1]. Mechanistic studies have revealed that each cluster possesses a parent of origin–specific differentially DNA-methylated region (DMR), which is established during gametogenesis and generally controls imprinted expression of part or all of the cluster and therefore is also termed the imprinting control region (ICR) [16].

High-throughput sequencing strategies (including RNA-sequencing [RNA-seq] and genome-wide DNA methylation sequencing tools) using reciprocal hybrids of two mouse strains have been employed to identify imprinted genes based on parental-specific expression patterns or regions containing parental-specific DNA methylation [17, 18]. Meanwhile, haploid embryonic stem cells (haESCs) with a single set of chromosomes from sperm efficiently support the generation of mice after injection into oocytes [19]. In combination with clustered regularly interspaced short palindromic repeats (CRISPR)-Cas9 (an efficient genome/epigenome-editing system established from bacteria), haESCs open new avenues for functional analyses of imprinted genes during early embryonic development [19–21]. Technical advances have enabled the discovery of imprinting, identification of imprinted genes, and understanding of the functions and mechanisms of imprinting. We propose that new techniques will further advance the progress of imprinting studies in the future.

## Embryological reconstruction strategies demonstrate critical roles for imprinted genes in embryonic development

AG and gynogenetic/PG embryos generated through nuclear transfer technology exhibited opposite phenotypes of developmental defects: AG embryos developed predominantly extraembryonic lineages and had poorly developed embryonic tissue; PG embryos developed predominantly embryonic tissue and had limited development of extraembryonic components [9, 10]. To further investigate the fate of PG or AG cells during fetal and postnatal development, chimeric mice were generated by combining PG or AG cells with normal embryos through embryo aggregation or blastocyst injection technologies [22–29]. Consistent with previous observations in uniparental embryos, chimeras generated by combining AG/PG and normal embryos produced PG cells that efficiently contributed to descendants of inner cell mass (ICM) but not trophectoderm (TE)-derived cells; chimeric AG cells contributed strongly to all TE-derived cells but rarely to ICM-derived cells [23, 29]. Interestingly, when AG or PG ICM cells were injected into normal blastocysts to produce chimeric embryos, the presence of AG cells significantly increased embryonic growth, whereas PG cells substantially reduced the size of chimeric embryos [27], likely because of the dosage changes of *Igf2*, a paternally expressed imprinted gene [14]. These observations support the parental conflict theory [30] and suggest that parental imprinting establishes the balance of gene dosage, which is critical for normal embryonic growth regulation.

Early nuclear transfer experiments demonstrated that genomic imprinting may impede the development of uniparental embryos and posed the intriguing question of whether the PG or AG embryos can develop to term when the dosage of imprinted genes is epigenetically or genetically manipulated. To test this, Kono and colleagues developed a serial nuclear transfer technology to construct PG embryos containing two sets of chromosomes from full grown (fg) oocytes with a normal maternal imprinted state and nongrowing (ng) oocytes in which de novo methylation of maternal-specific imprinting had not been established [31, 32]. They first fused an ng oocyte from a newborn mouse with an fg oocyte without the germinal vesicle (nucleus) to produce reconstructed oocytes, which were subjected to in vitro maturation to develop to metaphase II (MII, ng MII) stage. In the second nuclear transfer, the genome of ng MII oocytes was used as a donor for transfer into fg MII oocytes. The reconstructed oocytes were then parthenogenetically activated in activation medium without cytocholasin B, leading to exclusion of the two second polar bodies and formation of diploid PG embryos [31]. Using this technology, they showed that PG embryos containing ng oocytes with a single deletion of *H19* successfully developed to term and survived to adulthood with low efficiency [33] and later showed that PG embryos generated from ng oocytes with a double mutation of *H19*-DMR and intergenic germline-derived (*IG*)-DMR could develop into viable and fertile adults with a much higher efficiency, nearly comparable with that of in vitro fertilization [34]. The results provide direct evidence that these two paternally methylated ICRs are barriers to the normal development of PG embryos.

The generation of cloned animals through somatic cell nuclear transfer (SCNT) [35–38] provides new ways to study the function of imprinting during development. Successful SCNT in different species has demonstrated that adult somatic cells generally sustain genomic imprints that are established in the parental germline and stably maintained throughout embryonic development [38]. In contrast, imprint-free primordial germ cells are unable to support full-term development of cloned mouse embryos [39–41]. Similarly, mouse spermatogonial stem cells (SSCs) with a paternal imprinting state also failed to support cloned embryo development. Our preliminary data showed that none of the 2,068 cloned embryos from SSCs developed to term in vivo. These results strongly demonstrate that the correct imprinting state is critical for mouse embryonic development after SCNT. However, the birth rate of cloned mice is extremely low—approximately 2% of transferred embryos developed to term in surrogate mothers [36–38]. Interestingly, most cloned mice displayed an overgrowth of fetuses and placentas when cultured cells were used as donors, a phenotype referred to as “large offspring syndrome” [36, 42, 43], likely caused by epigenetic abnormalities, especially those in imprinted genes [42, 43]. In contrast, when mouse fresh or primary-cultured cells were used for cloning, the weight of the cloned placenta was consistently 2- to 3-fold above that of normal placenta, whereas most of the cloned pups were not oversized at birth [44–48]. Consistently, the imprinting state in the cloned pups mimicked that of the controls, whereas cloned placentas exhibited a significant reduction in expression of several imprinted genes compared with control placentas [48]. These results suggest that the imprinting state established during gametogenesis is stably maintained in somatic cells and not readily transmuted in enucleated oocytes [48]; imprinting loss in animals cloned from cultured cells probably happens in donor cells upon prolonged culturing [42, 43, 49]. Moreover, the abnormality consistently observed in cloned placentas from both freshly isolated and cultured cells implies that reprogramming errors—especially in imprinted genes—involved in placental function occur in extraembryonic lineages after nuclear transfer. This is consistent with recent observations from large-scale knockout phenotyping analysis, which show that placentas are preferentially vulnerable to defects after gene knockout [50], and likely explains the poor development of cloned embryos in vivo [37]. This hypothesis has been validated by the observation that cloning efficiency is significantly improved after replacement of the cloned placenta with a functional tetraploid placenta [51]; the cloned ICM is functionally equivalent to the fertilized ICM and may be a safe source of pluripotent cell derivation for therapeutic purposes [38]. Therefore, SCNT embryos provide a defective developmental model that can be used to investigate differences between cloned and fertilized embryos, which will not only aid our understanding of the underlying mechanisms of embryonic development following natural fertilization but may also provide the clues to improve the cloning efficiency for reproductive and therapeutic applications.

## Identification of imprinted genes using high-throughput analyses

Imprinted genes, differentially expressed from the maternal and paternal alleles, are controlled by an allele-specific differentially methylated region. Therefore, to screen differentially expressed genes or differentially methylated regions, the first challenge is to distinguish maternal transcripts or DNA methylation from the paternal allele. There are three main strategies used for this purpose [52, 53]: mouse strains carrying rearranged chromosomes including uniparental disomy or uniparental duplication of whole or specific chromosomal regions (generally produced by irradiation or chemical mutagenesis), Robertsonian translocations derived from wild populations, and specific arrangements generated by the Cre-loxP recombination system [52, 53]; PG or AG embryos/cells generated through embryo reconstitution; and filial (F)1 hybrids from two different mouse strains with the single-nucleotide polymorphisms (SNPs). These different approaches (divided here into three classes) have been employed to identify novel imprinted genes: gene expression (subtractive hybridization, differential display, microarray, and RNA-seq); epigenetic features (methylation representational difference analysis [Me-RDA], restriction landmark genomic scanning [RLGS], bisulfite sequencing and DNase-sequencing [DNase-seq]); and DNA sequence (computational analysis) (Table 1).

**Table 1:** Methods used to identify imprinted genes in mouse.

Screens based on	Advantages	Disadvantages	Techniques	Typical imprinted genes	References
Gene expression	Directly identify imprinted genes according to allele-specific expression	Limited to identification of expressed genes in specifically analyzed tissues and developmental stages	Subtractive hybridization	*Peg3*, *Peg1*, *Nnat*, *Meg1*, *Zac1*, *Sgce*	[54–58]
			Differential display	*Dlk1*, *Rian*, *Impact*	[59–61]
			Microarray	*Dlk1*, *Asb4*, *Ata3*, *Dcn*	[62]
			RNA-seq	*Inpp5f*, *Blcap*, *Casd1*, *NMIT3*, *Pde4d*, *Tbc1d12*, *Pde10*, *Phf17*, *Phactr2*, *Zfp64*, *Htra3*	[63, 64]
Epigenetic modification	Epigenetic modifications generally maintained throughout development	Directly identify imprinted domains rather than individual genes	RLGS	*Grf1*, *U2af1-rs1*	[65, 66]
			Me-RDA	*Nesp*, *Gnasxl*, *Nespas*, *Nap1l5*, *Peg13*, *Slc38a4*	[67, 68]
			Bisulfite sequencing	New DMRs	[17, 69]
			DNase-seq	*Smoc1*, *Epas1*, *Etv6*, *Otx2*, *Rbms1*, *Slc38a1*, *Slc38a2*	[70]
DNA sequence	Find candidates more efficiently in the whole genome	Need to be validated through experiments; may leave out imprinted genes with different sequence features	Bioinformatics analysis	*Mcts2*	[71]

Abbreviations: *Asb4*, ankyrin repeat and SOCS box containing 4; *Ata3*, amino acid transport system A3; *Blcap*, bladder cancer–associated protein; Casd1, CASD1 domain containing protein; *Dcn*, decorin; *Dlk1*, delta homolog 1; DMR, differentially DNA-methylated region; DNase-seq, DNase-sequencing; *Epas1*, endothelial PAS domain-containing protein 1; *Etv6*, ETS-variant gene 6; *Gnasxl*, guanine nucleotide‐binding protein, alpha stimulating extra‐large; *Grf1*, general regulatory factor 1; *Htra3*, HtrA serine peptidase 3; *Impact*, impact RWD domain protein; *Inpp5f*, inositol polyphosphate-5-phosphatase F; *Mcts2*, malignant T cell amplified sequence 2; Me-RDA, methylation representational difference analysis; *Meg1*, maternally expressed 1; *Nap1l5*, nucleosome assembly protein 1 like 5; *Nesp*, neuroendocrine secretory protein; *Nespas*, neuroendocrine secretory protein antisense; *Nnat*, neuronatin; *Otx2*, orthodenticle homebox 2; *Peg1*, paternally expressed 1; *Peg3*, paternally expressed 3; *Peg13*, paternally expressed 13; *Pde4d*, phosphodiesterase 4D; *Pde10*, phosphodiesterase type 10; *Phactr2*, phosphatase and actin regulator 2; *Phf17*, PHD finger protein 17; *Rbms1*, RNA binding motif single stranded interacting protein 1; *Rian*, maternally expressed 8; RLGS, restriction landmark genomic scanning; RNA-seq, RNA-sequencing; *Sgce*, epsilon-sarcoglycan; *Slc38a1*, solute carrier family 38 member 1; *Slc38a2*, solute carrier family 38 member 2; *Slc38a4*, solute carrier family 38 member 4; *Smoc1*, secreted modular calcium-binding protein 1; *Tbc1d12*, TBC1 domain family member 12; *U2af1-rs1*, U2 small nuclear ribonucleoprotein auxiliary factor 35 kDa subunit-related protein 1; *Zac1*, zinc finger 1; *Zfp64*, zinc finger protein 64

Systemic screening through subtractive hybridization of cDNA obtained from PG and normally fertilized embryos led to the identification of a series of paternally expressed genes (*Pegs*), including *Peg1*/mesoderm-specific transcript homolog protein (*Mest*), *Peg3*, and *Peg5* [54–56]. Using the same strategy to compare differential expression of genes between AG, PG, and normal embryos or fibroblasts, the maternally expressed gene (*Meg*) *Meg1*/ growth factor receptor bound protein 10 (*Grb10*) [57] and *Pegs*, zinc finger 1 (*Zac1*) and epsilon-sarcoglycan (*Sgce*) [58], were identified. Another systematic screen based on the allelic expression compared mRNA from two highly polymorphic mouse strains or that from uniparental and fertilized embryos and identified paternally expressed delta homolog 1 (*Dlk1*) gene [59] and maternally expressed 8 (*Rian*) [60].

With the rapid development of high-throughput technologies, novel imprinted genes were efficiently identified through large-scale comparison of gene expression between PG and AG embryos. Microarrays, which enable detection of the expression levels of thousands of genes at one time, have been used to successfully identify imprinted genes including ankyrin repeat and SOCS box containing 4 (*Asb4*), amino acid transport system A3 (*Ata3*), and decorin (*Dcn*) [62]. RNA-seq, which detects the whole transcriptome, has been employed to identify three new imprinted genes (*1810044A24Rik*, bladder cancer–associated protein [*Blcap*], and inositol polyphosphate-5-phosphatase F [*Inpp5f*]) in neonatal brains [63] and five new imprinted genes (phosphodiesterase type 10 [*Pde10*], PHD finger protein 17 [*Phf17*], phosphatase and actin regulator 2 [*Phactr2*], zinc finger protein 64 [*Zfp64*], and HtrA serine peptidase 3 [*Htra3*]) in embryonic day (E)17.5 placentae [64] from reciprocal F1 hybrids of two mouse strains with specific SNPs.

Gene expression–related screening strategies can only detect genes expressed in the analyzed tissues at a given developmental stage, whereas DMRs established in germ cells can be stably maintained in any tissue during development and provide a unique marker to identify imprinted genes. Gametic DMRs often control a cluster of imprinted genes, so methods based on DNA methylation can be used to screen an imprinted domain rather than a single imprinted gene. Me-RDA and RLGS—both of which use methylation-sensitive enzymes to identify and cut differentially methylated sequences between two samples—have been developed to identify novel imprinted genes [65–68, 72]. Additionally, high-throughput methylation analyses, such as whole-genome bisulfite sequencing (WGBS) and reduced representation bisulfite sequencing (RRBS) combined with bioinformatic analysis have been used to efficiently identify imprinted DMRs [17, 69]. However, if the parental origins of the alleles are heterozygous tissues in which a monoallelically expressed gene is identified by RNA-seq and DNA-seq, one cannot conclude that the gene is imprinted, because *Cis*-expression quantitative trait loci (*Cis*-eQTL) and RNA editing are potentially confounding.

In addition to screening strategies that rely on parent of origin–specific transcription and epigenetic marks, computational methods based on DNA sequence similarity shared in known imprinted genes (CpG islands, noncoding RNA, and repeat elements) have also been used to predict and identify new imprinted genes, generally combined with experimental validation [71, 73, 74].

## Replacement of gamete genome with haESCs for imprinting studies

**Fig 1:**
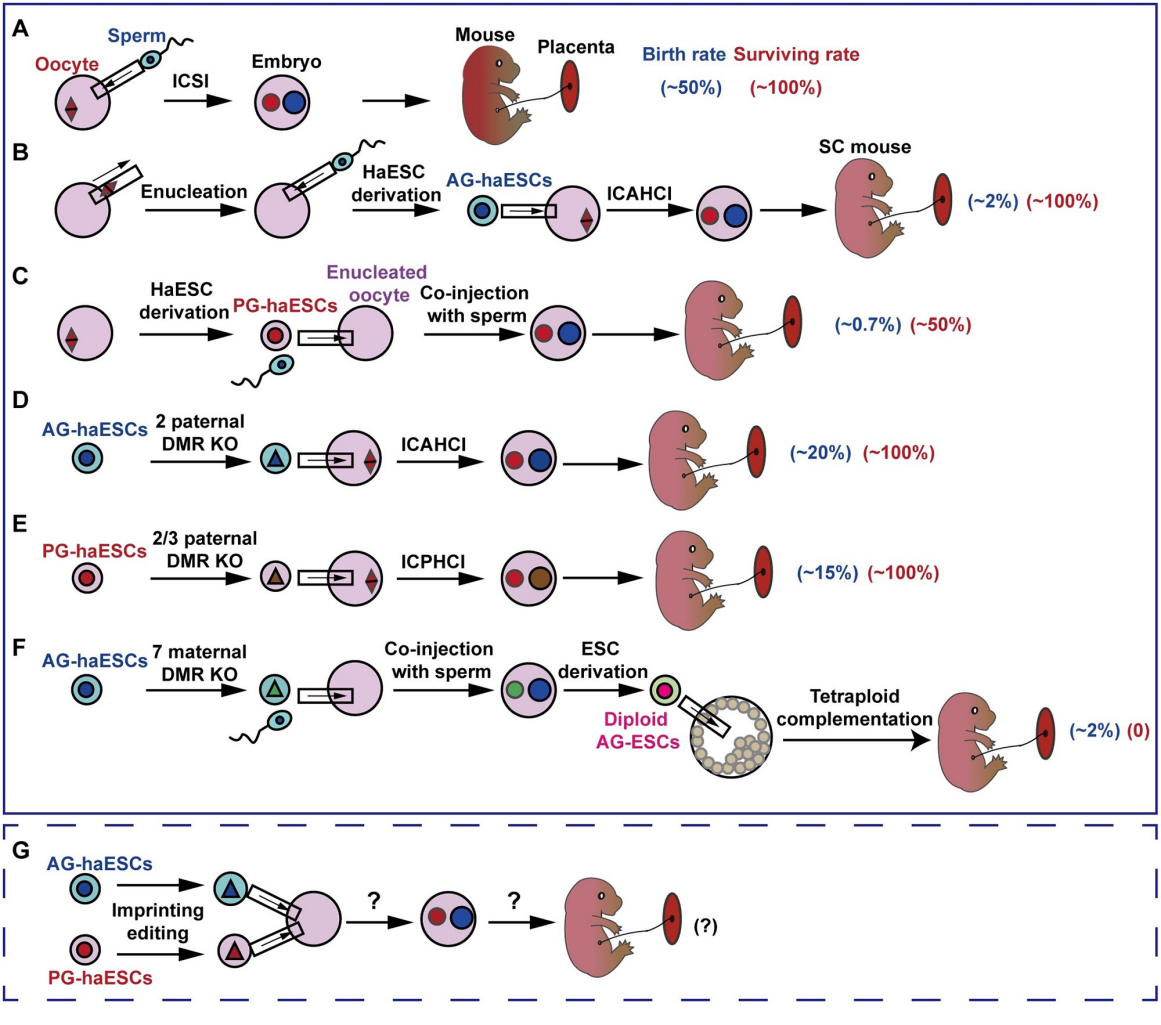
Generation of normal mice using haESCs as gamete replacements. (A) ICSI can generate normal mice. (B) The AG-haESCs support full-term embryonic development through ICAHCI, but with very low developmental efficiency. The generated mice are termed SC mice. (C) The PG-haESCs support full-term embryonic development through coinjection with sperm into enucleated oocytes, but with very low developmental efficiency. (D and E) The AG or PG-haESCs with deletions of two or three paternal DMRs efficiently generate normal mice through ICAHCI or ICPHCI. (F) The sperm-originated AG-haESCs with deletions in seven maternally imprinted regions (*Nespas*, *Peg3*, *Snrpn*, *Kcnq1*, *Grb10*, *Igf2r*, and *Gnas*) can be used to replace oocyte genomes for full-term development of bipaternal embryos using tetraploid complementation technology. (G) It is not clear whether AG-haESCs and PG-haESCs cultured in vitro can simultaneously substitute for paternal and maternal genomes and generate normal mice. The experiments that have been performed are outlined with a solid box; proposed experiments are outlined with a dashed box. Birth rate: percentage of transferred embryos. Surviving rate: percentage of born pups. AG, androgenetic; DMR, differentially DNA-methylated region; ESC, embryonic stem cell; *Grb10*, growth factor receptor bound protein 10; haESC, haploid embryonic stem cell; ICAHCI, intracytoplasmic AG-haESC injection; ICPHCI, intracytoplasmic PG-haESC injection; ICSI, intracytoplasmic sperm injection; *Igf2r*, insulin-like growth factor 2 receptor; *Kcnq1*, potassium voltage-gated channel subfamily Q member 1; KO, knockout; *Nespas*, neuroendocrine secretory protein antisense; *Peg3*, paternally expressed 3; PG, parthenogenetic; SC, semicloned; *Snrpn*, small nuclear ribonucleoprotein-associated protein N.

The functional analysis of imprinted genes in vivo relies heavily on germline transmission of gene knockouts of parental-specific alleles. However, both oocytes and sperm are structurally specialized cells that cannot be genetically manipulated in vitro or in vivo. The standard gene-targeting approach based on homologous recombination in diploid ESCs, although successfully used for the last 3 decades, is time-consuming and labor-intensive and has significant limitations including the necessity of germline transmission, which is typically the rate-limiting step [75, 76]. To overcome these challenges, scientists sought to use haploid cells, generated from either sex, in place of the oocyte/sperm genome to produce animal models [77]. In 2011, nearly 40 years after the first efforts to generate haploid embryos and cells [78, 79], mouse PG-haESCs were successfully established from PG haploid blastocysts carrying only one set of chromosomes from oocytes; the haploid state was maintained through regular enrichment using fluorescence-activated cell sorting (FACS) during long-term in vitro culture [80, 81]. One year later, two separate research groups derived mouse AG-haESCs from reconstructed embryos containing only sperm-derived chromosomes through injection of sperm into enucleated oocytes or removal of female pronuclei from zygotes [19, 82]. Strikingly, except for the haploidy and pluripotency that are the typical features of PG-haESCs, AG-haESCs also generally maintain paternal genomic imprints and can be used as a sperm replacement to support full-term development upon injection into mature oocytes (intracytoplasmic AG-haESC injection [ICAHCI]) [19] (Fig 1B). The resulting animals are referred to as semicloned (SC) mice because one-half of their genomes is from the cultured cells and the other is from different oocytes [19]. Importantly, the AG-haESCs enabled in vitro genetic manipulation, thus providing new methods for the efficient production of genetically modified mice [21]. The birth rate of SC mice was extremely low, with only approximately 4% of the transferred embryos developing to term, and half of those exhibiting a growth-retarded phenotype and dying shortly after birth [19]. When the oocyte genome was replaced with that from PG-haESCs, the reconstructed SC embryos developed to term with extremely low efficiency (0.7% of transferred embryos) [83] (Fig 1C). We reasoned that the developmental failure of SC embryos was likely caused by global DNA demethylation (including in imprinted DMRs) of haESCs induced by 2i culture medium [84]. We then demonstrated that loss of DNA methylation in *H19* and *IG*-DMRs that control the expression of *Igf2/H19* and *Dlk1/*iodothyronine deiodinase 3 (*Dio3*), respectively, led to the dysregulation of imprinted genes and growth retardation in embryos [85]. When the *H19*-DMR and *IG*-DMR were removed in AG-haESCs, the resulting cells (double knockout [DKO]-AG-haESCs) could efficiently support SC mice generation, with a birth rate of approximately 20% of transferred embryos [85] (Fig 1D). Importantly, after multiple rounds of gene modifications in vitro, these cells could still efficiently produce healthy mice (which contained the corresponding genetic traits) in a single-step process through ICAHCI [85]. Thus, DKO-AG-haESCs (termed “artificial spermatids”) are a feasible tool for spermatid replacement in the efficient generation of mice with complex gene modifications.

Previous observations in bimaternal mice generated from fusion of fg oocytes with ng oocytes carrying *H19*-DMR and *IG*-DMR deletions [34], combined with the observation that *H19*-DMR and *IG*-DMR are barriers to stable developmental of AG-haESCs, led us to reason that correct expressions of *Igf2/H19* and *Dlk1/Dio3* loci are critical for the haESC genome to be used as a sperm replacement for embryonic growth in mice. Consistent with this hypothesis, we and others confirmed that oocyte-originated PG-haESCs carrying both *H19*-DMR and *IG*-DMR deletions could be used to efficiently generate live mice upon injection into oocytes [86, 87] (Fig 1E). Most recently, sperm-originated AG-haESCs with deletions in seven maternally imprinted regions known to affect embryogenesis (neuroendocrine secretory protein antisense [*Nespas*], *Peg3*, small nuclear ribonucleoprotein-associated protein N [*Snrpn*], potassium voltage-gated channel subfamily Q member 1 [*Kcnq1*], *Grb10*, *Igf2r*, and *Gnas*) were used to replace the genome of oocytes for full-term development of bipaternal embryos [88] (Fig 1F), further suggesting that maternally imprinted loci may also be major barriers to the ability of haESCs to replace the oocyte genome. However, only two live-born bipaternal pups were obtained, and both died in 2 days, suggesting that additional critical imprinted genes need to be manipulated in AG-haESCs to enhance the fetal and postnatal development of these embryos. Taken together, imprinting is indeed the specific epigenetic feature of oocyte and sperm genome critical for normal embryonic development; haESCs with correct expression levels of imprinted genes (generated by genetic modifications) may be used to replace both parental genomes for normal embryonic development (Fig 1G).

## Functional validation of imprinted genes through genome-editing strategies

For over 30 years, the homologous recombination-based “knockout” method has been the standard approach to determine the function of imprinted genes in vivo [75, 76]; loss of imprinting (LOI) knockout models reveal specific functional effects of imprinted genes [89]. However, the conventional knockout strategy is time and labor consuming and greatly limits the progress of functional analyses of imprinted genes. Recently, the RNA-guided CRISPR-associated Cas9 endonucleases, developed from the microbial adaptive immune system, has been widely applied as a highly versatile and efficient tool for genome editing in diverse organisms [90–93]. CRISPR-Cas9–based genome editing requires a single guide RNA (sgRNA) to direct Cas9 cleavage at a specific site; cleavage is followed by stimulation of nonhomologous end joining (NHEJ) or homology-directed repair (HDR)-mediated genome editing and results in precise and efficient genome editing [94–97]. Through direct injection into zygotes, CRISPR-Cas9 was used to efficiently delete the large imprinted *Rian* long noncoding RNA (lncRNA) in vivo, which abolished the expression of the corresponding *Rian* gene from the maternally inherited allele [98], a manipulation that has been extremely difficult to achieve using conventional methods. A nuclease-deactivated form of Cas9 (dCas9), which lacks the nuclease activity but retains its RNA-guided DNA-binding activity [99], acts as a scaffold to recruit other effectors to mediate site-specific transcriptional modulation or epigenetic regulation without changing genomic content [90]. Fusion of dCas9 with DNA methyltransferase 3A (DNMT3A) enables the addition of DNA methylation marks at a specific site, offering a rational epigenome-editing strategy for correction of aberrant imprinting in human disorders [100, 101].

## Perspective: The combined application of haESCs and CRISPR-Cas9 may dramatically enhance functional analyses of imprinted genes in vivo

**Fig 2:**
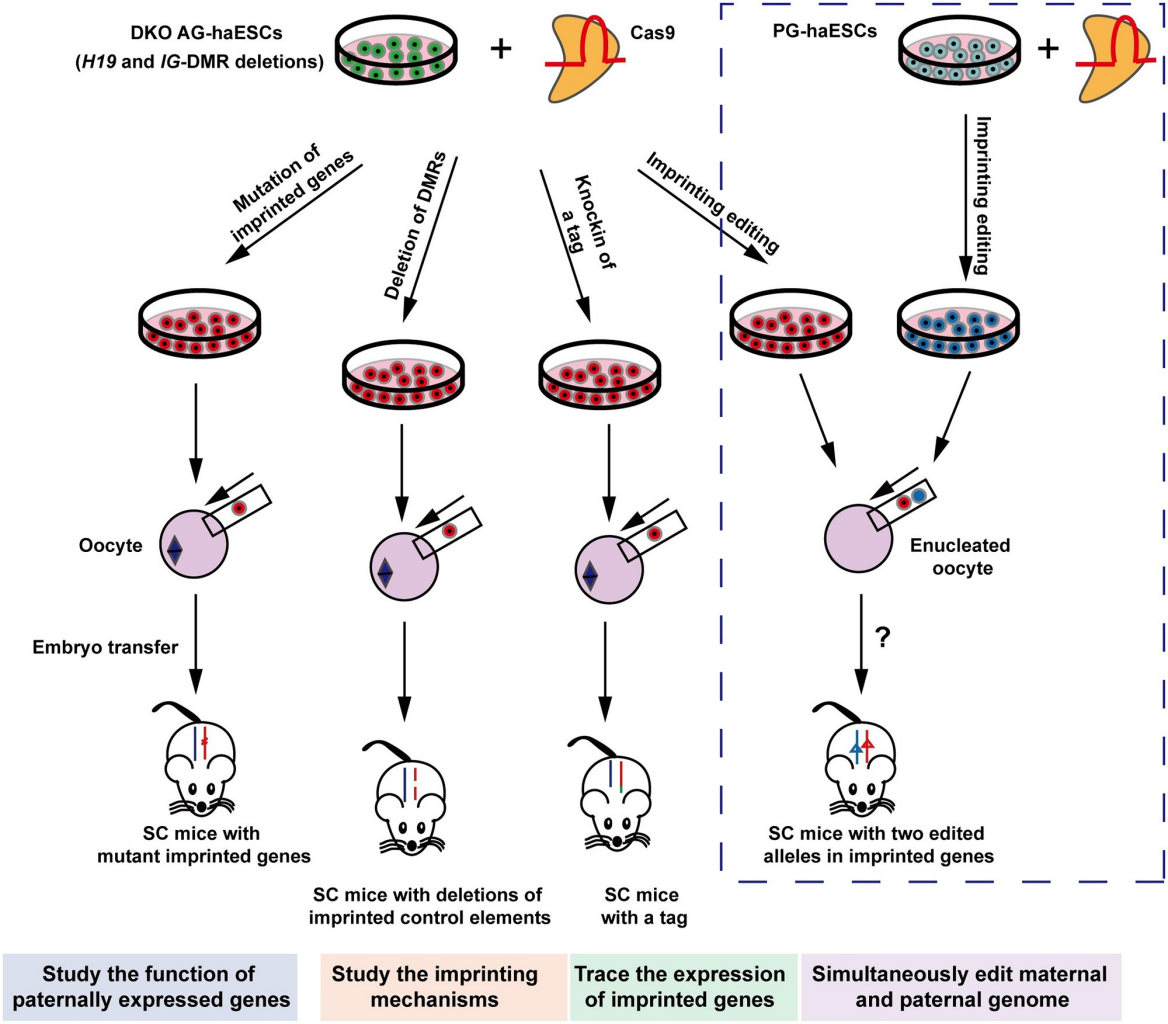
Combined applications of haESCs and CRISPR-Cas9 in functional studies of imprinted genes in vivo. The sperm-originated AG-haESCs carrying deletions in both *H19*-DMR and *IG*-DMR combined with CRISPR-Cas9 editing technology can be used to study the function of paternally expressed genes and imprinting mechanisms and to trace the expression of imprinted genes. If AG-haESCs and PG-haESCs can be used to substitute for maternal and paternal genomes, respectively, and support normal embryonic development, it may be possible to simultaneously edit maternal and paternal alleles of the imprinted gene in the future (labeled by dashed box). AG, androgenetic; DKO, double knockout; DMR, differentially DNA-methylated region; H19, a long noncoding RNA; haESC, haploid embryonic stem cell; IG, intergenic germline-derived; PG, parthenogenetic, SC, semicloned.

HaESCs have been successfully derived from different species, including rat [102], monkey [103], and human [104, 105]. HaESCs facilitate genetic analyses of mammals at the cellular level because they contain only one set of chromosomes. Importantly, mouse AG-haESCs with *H19*-DMR and *IG*-DMR deletions can be used for efficient and stable generation of SC mice through ICAHCI. These “artificial spermatids” can be used to make multiple genetic modifications in vitro using CRISPR-Cas9 technology and generate mouse models with new genetic characteristics in a single step [20, 21, 106–108]. This combined application provides a novel genetic tool for functional analysis of the imprinted gene in vivo (Fig 2). One of the potential applications is to quickly validate candidate maternally imprinted genes through high-throughput analyses by mutating the candidate gene in “artificial spermatids.” We postulate that if the maternally imprinted gene is essential for embryonic development, deletion of the gene in “artificial spermatids” would result in SC embryos without its expression during the entire process of embryonic development and would most likely lead to developmental abnormalities. Control elements for a specific imprinted gene could be easily removed or modified in “artificial spermatids” to produce mouse models to investigate the mechanism of imprinting. For example, deletion of different regions across X-inactive specific transcript (*Xist*) locus [109, 110] in “artificial spermatids” may facilitate the identification of the functional elements of *Xist* that regulate X chromosome inactivation during mouse embryo development. In addition, genetically labeling an imprinted gene with a fluorescent or affinity tag in “artificial spermatids” would result in the ability to visualize, localize, and identify protein interactions in tagged mice in vivo [111]. Moreover, haESCs with mutations in imprinted genes could be used to replace maternal genomes to support the full-term embryonic development; however, because the current efficiency of the technique is extremely low, future work is required to determine whether other maternal methylation imprints need to be genetically manipulated in haESCs to improve their oocyte-like features. Finally, it is still unclear whether haESCs could be used to replace the genomes of both oocytes and sperm for efficient full-term embryonic development (Fig 2). We propose that if both paternal and maternal genomes could be replaced by haESCs, it would be very convenient to simultaneously edit maternal and paternal genomes and facilitate the study of imprinted gene function and regulatory mechanisms in vivo.
